# Supplementary material for: Impact of Isoquinoline Alkaloids on the Intestinal Barrier in a Colonic Model of Campylobacter jejuni Infection
Source: Int J Mol Sci. 2025 Oct 31;26(21):10634. doi: 10.3390/ijms262110634 (PMC12609152; doi:10.3390/ijms262110634)
Supplement: Supplementary file 1 [file ijms-26-10634-s001.zip › ijms-3948422-supplementary.pdf]

Table S1. Brain Heart Infusion Broth (BHI) (https://pol-aura.pl/brain-heart-infusion-broth-p-26639.html)

Composition	Volume [g/L]
HM infusion powder (equivalent to calf brain infusion from)	12.5
BHI powder (equivalent to beef heart infusion from)	5.0
Proteose peptone	10.0
Dextrose (glucose)	2.0
Sodium chloride	5.0
Disodium phosphate	2.5
Final pH (at 25°C): 7.4±0.2	

Table S2. Columbia agar (CA) (https://pol-aura.pl/columbia-blood-agar-base-p-26547.html)

Composition	Volume [g/L]
Special peptone	23.0
Starch	1.0
Sodium chloride	5.0
Agar	10.0
Final pH (at 25°C): 7.3±0.2	

Table S3. Formulation for Eagle's Minimum Essential Medium (EMEM) ATCC 30-2003 (https://www.atcc.org/products/30-2003)

Composition	Volume
**Inorganic Salts [g/L]**	
CaCl2 (anhydrous)	0.2
KCl	0.4
MgSO4 (anhydrous)	0.09767
NaHCO3	1.5
NaH2PO4·H2O	0.14
NaCl	6.8
**Amino Acids [g/L]**	
L-Alanine	0.00890
L-Arginine·HCl	0.12640
L-Asparagine·H2O	0.01500
L-Aspartic Acid	0.01330
L-Cystine·2HCl	0.03120
L-Glutamic Acid	0.01470
L-Glutamine	0.29200

Glycine	0.00750
L-Histidine·HCl·H2O	0.04190
L-Isoleucine	0.05250
L-Leucine	0.05250
L-Lysine·HCl	0.07250
L-Methionine	0.01500
L-Phenylalanine	0.03250
L-Proline	0.01150
L-Serine	0.01050
L-Threonine	0.04760
L-Tryptophan	0.01000
L-Tyrosine·2Na·2H2O	0.05190
L-Valine	0.04680
**Vitamins [g/L]**	
Choline Chloride	0.00100
Folic Acid	0.00100
myo-Inositol	0.00200
Nicotinamide	0.00100
D-Pantothenic Acid (hemicalcium)	0.00100
Pyridoxine·HCl	0.00100
Riboflavin	0.00010
Thiamine·HCl	0.00100
**Other [g/L]**	
D-Glucose	1.00000
Phenol Red, Sodium Salt	0.01000
Sodium Pyruvate	0.11000

Table S4. Characteristics of Fed State Artificial Colonic Fluid (https://biochemazone.com/product/fed-state-artificial-colonic-fluid-fesac-bz380/?srsltid=AfmBOorIgPV2YD3q7Z5hxnaDdpAdCQWdODHcARlrTnjM91Kuh8GnZPT3)

Composition	Volume
Physiological components: bile acid and lipids	unknown
Tris	unknown
Malei acid	unknown
Minerals: sodium and chloride	unknown
Phosphate	unknown
pH: 6.0	

Figure S1. Viability of *C. jejuni* after 72-h exposure to MIC values of different isoquinoline alkaloids: A – control, B – SAN, C – CHE, D – BBR. Bacteria were stained with LIVE/DEAD

staining kit. Scale bar, 20 μm

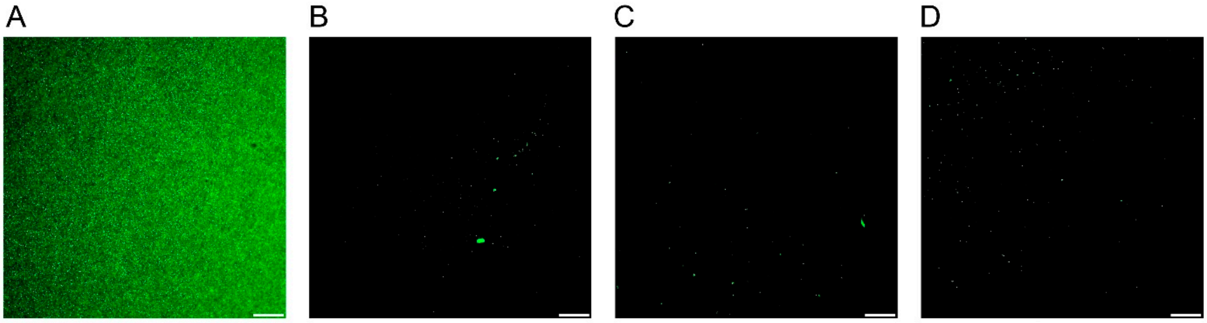

Table S5. Mean cell viability of CCD841 CoN cells after 24-h exposure to isoquinoline alkaloids in the MTT assay. Values are expressed as mean ± SD (%) from n = 4 independent replicates per concentration.

Compound	Concentration	Mean Cell Viability	SD
BBR	32	93.80	8.186
	64	95.77	3.037
	128	101.36	1.197
CHE	16	31.98	3.543
	32	22.44	1.375
	64	23.45	0.332
SAN	8	24.82	1.207
	16	24.09	1.355
	32	24.58	1.070

Table S6. Pairwise Wilcoxon rank-sum tests comparing concentrations within each compound (BBR, CHE, SAN) in the MTT assay. For each compound, n = 4 per concentration; three pairwise dose comparisons were performed and Bonferroni-adjusted. The table reports raw and adjusted p-values with significance (ns = not significant).

Compound	Group 1	Group	n_1	n_2	p-value	Adjusted p-	Significance
	32	64	4	4	1.000	1.000	ns
BBR	32	128	4	4	0.057	0.171	ns
	64	128	4	4	0.029	0.086	ns
	16	32	4	4	0.029	0.088	ns
CHE	16	64	4	4	0.029	0.088	ns
	32	64	4	4	0.306	0.918	ns
	8	16	4	4	0.686	1.000	ns
SAN	8	32	4	4	0.770	1.000	ns
	16	32	4	4	0.882	1.000	ns

Table S7. Pairwise Wilcoxon rank-sum tests comparing compounds (BBR, CHE, SAN) in the MTT assay, with per-cell viabilities pooled across concentrations. For each pair, sample sizes

(n_1, n_2) are the numbers of cell-level observations per compound; three pairwise comparisons were performed and Bonferroni-adjusted. The table reports raw and adjusted p-values with significance (ns = not significant; * p<0.05; ** p<0.01; *** p<0.001.).

Group 1	Group 2	n_1	n_2	p-value	Adjusted p-value	Significance
BBR	CHE	12	12	3.63×10^{-5}	1.09×10^{-4}	***
BBR	SAN	12	12	3.59×10^{-5}	1.08×10^{-4}	***
CHE	SAN	12	12	0.643	1	ns

Table S8. Pairwise Wilcoxon rank-sum tests comparing biofilm formation between culture conditions. Two-sided Wilcoxon tests were performed for all 15 pairs. Each condition had n = 12 observations (4 independent experiments × 3 technical repeats). *p*-values were Bonferroni-adjusted across the 15 comparisons; the table reports raw *p* and adjusted *p* ($p_{(Bonf)}$) with significance codes: ns (not significant, $p_{(Bonf)} \geq 0.05$), * ($p_{(Bonf)} < 0.05$), ** ($p_{(Bonf)} < 0.01$), *** ($p_{(Bonf)} < 0.001$), **** ($p_{(Bonf)} < 0.0001$). All +BBR vs matched BBR-free media were significantly different with $p_{(Bonf)} \leq 1.11\times10^{-5}$; DMEM vs MediumMix comparison was not significant ($p_{(Bonf)} = 1$).

Group 1	Group 2	n_1	n_2	p-value	Adjusted p-value	Significance
BHI5	BHI5+BBR		12	127.4×10^{-7}	0.0000111	****
BHI5	EMEM10		12	127.4×10^{-7}	0.0000111	****
BHI5	DMEM+BBR		12	127.4×10^{-7}	0.0000111	****
BHI5	E/Amix		12	127.4×10^{-7}	0.0000111	****
BHI5	E/Amix+BBR		12	127.4×10^{-7}	0.0000111	****
BHI5+BBR	EMEM10		12	121.48×10^{-6}	0.0000222	****
BHI5+BBR	EMEM10+BBR		12	127.4×10^{-7}	0.0000111	****
BHI5+BBR	E/Amix		12	127.4×10^{-7}	0.0000111	****
BHI5+BBR	E/Amix+BBR		12	127.4×10^{-7}	0.0000111	****
EMEM10	EMEM10+BBR		12	127.4×10^{-7}	0.0000111	****
EMEM10	E/Amix		12	120.291	1.000	ns
EMEM10	E/Amix+BBR		12	127.4×10^{-7}	0.0000111	****
EMEM10 +BBR	E/Amix		12	127.4×10^{-7}	0.0000111	****
EMEM10 +BBR	E/Amix+BBR		12	128.88×10^{-6}	0.000133	***
E/Amix	E/Amix+BBR		12	127.4×10^{-7}	0.0000111	****

Table S9. Kruskal–Wallis tests comparing epithelial monolayer surface coverage (%) among four groups (control, BBR, BBRCamp, Camp) at each time point (24, 48, 72, 96 h). Reported p-values are for the overall group comparison at each time. Significance codes: * p < 0.05; ns = not significant.

Time	n	df	p-value	Significance
24	12	3	0.0862	ns
48	12	3	0.0862	ns

72	12	3	0.0444	*
96	12	3	0.0329	*

Table S10. Pairwise Wilcoxon rank-sum tests comparing cell-size distributions between control, BBR, BBRCamp, and Camp (cells pooled across biological replicates I–III). Bonferroni correction was applied within the six pairwise comparisons. The table reports sample sizes (n_1, n_2), raw *p*-values, and Bonferroni-adjusted *p*-values with significance codes. Camp differed from all other groups ($p_{(Bonf)}$ ranged from $1.99{\times}10^{-19}$ to $8.28{\times}10^{-17}$), whereas control vs BBR, control vs BBRCamp, and BBR vs BBRCamp were ns (not significant, $p_{(Bonf)}$ = 1).

Group 1	Group 2	n1	n2	p-value	Adjusted p-value	Significance
BBR	Camp	672	247	1.38e-17	8.28×10^{-17}	****
BBRCamp	Camp	540	247	3.31e-20	1.99×10^{-19}	****
Camp	control	247	662	2.02e-18	1.21×10^{-17}	****
BBR	BBRCamp	672	540	8.8e-01	1	ns
BBR	control	672	662	4.56e-01	1	ns
BBRCamp	control	540	662	2.25e-01	1	ns

Table S11. Summary statistics for barrier permeability of CCD841 CoN monolayers measured as % Lucifer Yellow (LY) passage at 24h, 48h, 72h, and 96h across four conditions: control, BBR, BBRCamp (BBR + *C. jejuni* post-culture supernatant), and Camp (*C. jejuni* supernatant). Values are mean ± SD; n denotes the number of replicates contributing to each estimate (after the IQR-based outlier screen used in the analysis).

Time	Group	n	Mean ± SD
24h	control	7	15.1 ± 0.18
24h	BBR	6	14.1 ± 0.16
24h	BBRCamp	7	13.1 ± 0.81
24h	Camp	6	16.0 ± 0.37
48h	control	6	12.4 ± 0.35
48h	BBR	6	11.0 ± 0.18
48h	BBRCamp	7	11.6 ± 0.17
48h	Camp	7	13.8 ± 0.21
72h	control	7	11.3 ± 0.09
72h	BBR	7	10.7 ± 0.12
72h	BBRCamp	7	11.8 ± 0.64
72h	Camp	6	13.7 ± 0.10
96h	control	5	11.6 ± 0.03
96h	BBR	7	10.8 ± 0.21
96h	BBRCamp	7	12.2 ± 0.34
96h	Camp	7	15.1 ± 0.29

Table S12. Pairwise Wilcoxon rank-sum tests with Bonferroni correction comparing Lucifer Yellow permeability of CCD841 CoN monolayers across control, BBR, BBRCamp (BBR + *C. jejuni* post-culture supernatant), and Camp (*C. jejuni* supernatant) at 24h, 48h, 72h, and 96h. Reported are raw and adjusted *p*-values. Significance codes: * (adjusted $p < 0.05$), ns (not significant).

Time		24h		48h		72h		96h	
**Group 1**	**Group 2**	**p-value**	**Adjusted p-value**	**p-value**	**Adjusted p-value**	**p-value**	**Adjusted p-value**	**p-value**	**Adjusted p-value**
BBR	BBRCamp	0.038	0.230(ns)	0.003	0.020(*)	0.005	0.030(*)	0.002	0.013(*)
BBR	Camp	0.005	0.030(*)	0.003	0.020(*)	0.003	0.020(*)	0.002	0.013(*)
BBR	control	0.003	0.020(*)	0.005	0.030(*)	0.002	0.013(*)	0.006	0.034(*)
BBRCamp	Camp	0.003	0.020(*)	0.002	0.013(*)	0.003	0.020(*)	0.002	0.013(*)
BBRCamp	control	0.002	0.013(*)	0.003	0.020(*)	0.250	1.000(ns)	0.023	0.135(ns)
Camp	control	0.005	0.032(*)	0.003	0.020(*)	0.003	0.020(*)	0.006	0.034(*)

Figure S2. The effect of occludin relocation in CCD841 CoN colonocytes caused by the presence of *C. jejuni* in the environment. Time point: 96 hours. A - control, B - BBR, C - BBRCamp, D - Camp. Staining: rabbit polyclonal anti-occludin antibodies conjugated with fluorescent dye CoraLite®594 at a dilution of 1:250. Scale bar, 120 μm

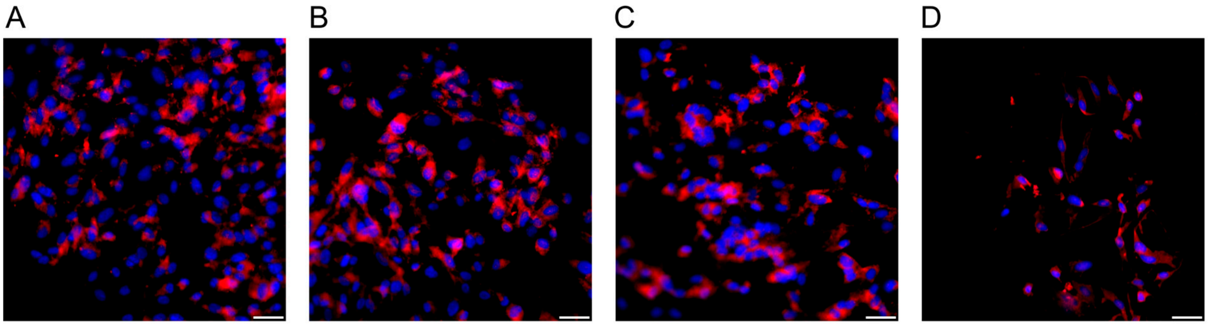

Table S13. Kruskal–Wallis tests with Benjamini–Hochberg FDR correction comparing metabolite levels across 12 experimental groups: control, BBR, BBRCamp (BBR + *C. jejuni* post-culture supernatant), and Camp (*C. jejuni* supernatant) at 0h, 48h, and 96h. Reported are raw and adjusted p-values. Significance codes: * ($p < 0.05$), ** ($p < 0.01$), ns (not significant).

Metabolite name	p-value	Adjusted p-value	Significance
1-Methylnicotinamide (+)	4.6×10^{-4}	0.007	**
Glutamic acid (−)	8.29×10^{-4}	0.007	**
Glutamine (−)	6.61×10^{-4}	0.007	**
L-Pyroglutamic acid (+)	5.26×10^{-4}	0.007	**
LPC 18:1 (−)	6.83×10^{-4}	0.007	**
LPC 18:2 (−)	4.22×10^{-4}	0.007	**

N-Alpha-Acetyl-Ornithine (−)	8.46×10^{-4}	0.007	**
Urocanic acid (+)	8.38×10^{-4}	0.007	**
Arginine (−)	0.0016	0.007	**
Cholate (−)	0.0013	0.007	**
Citraconic acid (−)	9.8×10^{-4}	0.007	**
LysoPC (0:0/18:0) (+)	0.0012	0.007	**
PC (16:0/0:0) (+)	0.0016	0.007	**
Streptomycin (+)	0.0016	0.007	**
Vanillic acid (+)	0.0011	0.007	**
1-Stearoyl-sn-glycero-3-phosphocholine (+)	0.0018	0.007	**
N-epsilon-Acetyllysine (−)	0.0018	0.007	**
LPC 16:0 (−)	0.0020	0.008	**
Berberine (+)	0.0023	0.008	**
Citric acid (−)	0.0031	0.009	**
Glycocholate (−)	0.0031	0.009	**
L-Carnitine (+)	0.0027	0.009	**
LPC 18:3; PlaSMA ID-2742 (+)	0.0029	0.009	**
Streptomycin (−)	0.0028	0.009	**
4-Hydroxy-4-(pyridin-2-yl)butan-2-one (+)	0.0038	0.010	*
Tryptophan (−)	0.0038	0.010	*
N-Acetylhistidine (+)	0.0040	0.010	*
Arginine (+)	0.0043	0.010	*
Threonine (−)	0.0044	0.010	*
3-Methyl-2-oxindole (+)	0.0056	0.012	*
Oxypurinol (−)	0.0054	0.012	*
Serine (−)	0.0056	0.012	*
Tauroallocholic acid (−)	0.0053	0.012	*
D-(+)-Galactose / Hexose (−)	0.0065	0.013	*
L-Pyroglutamic acid (−)	0.0072	0.014	*
Carnosine (−)	0.0078	0.015	*
Sulfanilic acid (+)	0.0086	0.016	*
Lysine (−)	0.0092	0.017	*
Arachidonic acid (−)	0.0118	0.020	*
Isoleucine/Leucine (−)	0.0119	0.020	*
Taurochenodesoxycholic acid (−)	0.0120	0.020	*
PE 36:1 (−)	0.0144	0.024	*
PC 33:1 (−)	0.0174	0.028	*
Allantoin (−)	0.0186	0.029	*
2,3-Dideoxyuridine (−)	0.0214	0.033	*
Betaine (+)	0.0240	0.036	*
PC 35:1 (−)	0.0296	0.043	*
Tauroursodeoxycholic acid (+)	0.0363	0.052	ns
Phenylacetic acid (−)	0.0448	0.063	ns
Xanthosine (−)	0.0469	0.065	ns
Docosahexanoic acid (−)	0.0541	0.073	ns

Lysine (+)	0.0574	0.076	ns
PC (16:0/18:1(9Z)) (+)	0.0664	0.086	ns
PI 38:4 (−)	0.0774	0.099	ns
Histidine (+)	0.0843	0.106	ns
Alpha-oxo-1h-indole-3-propanoic acid (+)	0.136	0.168	ns
Lauryldiethanolamine (+)	0.159	0.192	ns
Deoxycholate (−)	0.173	0.206	ns
Phenylalanine (−)	0.190	0.222	ns
Vitamin B1 (+)	0.215	0.247	ns
Cycloserine_major (+)	0.227	0.253	ns
Quinolinic acid (−)	0.227	0.253	ns
Uridine (−)	0.237	0.260	ns
Hypoxanthine (+)	0.254	0.272	ns
N,N-Dimethylarginine (+)	0.256	0.272	ns
Phenylalanine (+)	0.282	0.295	ns
3-Guanidinopropionic acid (+)	0.516	0.531	ns
Lauryl sulfate (−)	0.701	0.711	ns
Phenylacetic acid + 2O, O-Hex (−)	0.803	0.803	ns
